# The Transformative Power of Virtual Hospitals for Revolutionising Healthcare Delivery

**DOI:** 10.3389/phrs.2024.1606371

**Published:** 2024-06-19

**Authors:** Alexandre Vallée, Maxence Arutkin

**Affiliations:** ^1^ Department of Epidemiology and Public Health, Foch Hospital, Suresnes, France; ^2^ School of Chemistry, Center for the Physics and Chemistry of Living Systems, Tel Aviv University, Tel Aviv-Yafo, Israel

**Keywords:** virtual hospital, healthcare, personalised medicine, digital health, health systems

## Abstract

**Objectives:** The objective of this narrative review is to explore the advantages and limitations of VHs in delivering healthcare, including access to specialized professionals, streamlined communication, efficient scheduling, integration of electronic health records, ongoing monitoring, and support, transcending geographical boundaries, and resource optimization.

**Methods:** Review of literature.

**Results:** The national healthcare systems are facing an alarming rise in pressure due to global shifts. Virtual hospitals (VH) offer a practical solution to numerous systemic challenges, including rising costs and increased workloads for healthcare providers. VH also facilitate the delivery of personalized services and enable the monitoring of patients beyond the conventional confines of healthcare settings, reducing the reliance on waiting medicine carried out in doctors' offices or hospitals.

**Conclusion:** VH can mirror the conventional healthcare referral system.

## Introduction

The national healthcare systems are facing an alarming rise in pressure due to global shifts in demographics, society, and epidemiology. This escalating pressure jeopardises their sustainability even in the near future. When considering the demand side, the primary factors contributing to this issue are the global population’s progressive aging with chronic diseases. By 2050, the population over the age of 60 is expected to grow from 1 billion in 2020 to 2.1 billion [1]. Additionally, there has been a consistent increase in the prevalence of chronic noncommunicable diseases in recent years, responsible for nearly 75% of deaths in Europe and 70%–80% of healthcare resource consumption worldwide. These diseases are the leading cause of death in the 54 countries of WHO Europe [2].

Addressing these changing needs and expressed demands necessitates the exploration of new organisational solutions. A shortage of healthcare professionals is a structural problem that, based on pre-pandemic data, could potentially reach 10 million worldwide by 2030 [3]. This shortage, coupled with an increased workload in healthcare facilities, has negatively impacted staff retention and the attractiveness of health professions [4]. This vicious cycle has led to an ongoing scarcity of healthcare personnel in both the medical and nursing fields.

The cost of national healthcare services in more developed countries has been on a continuous rise. Between 2013 and 2019, the annual *per capita* expenditure growth, adjusted for inflation, averaged 3% across EU member states [5]. This trend is significantly influenced by the cost of innovative pharmaceuticals in the market and investments in new technologies [5].

To address the critical issues mentioned above, technology and various digital tools have been increasingly utilised over the last two decades [6]. This includes smart and wearable medical devices for patients, which enable the remote delivery of healthcare services [7, 8]. This digital shift has been stimulated by the growing confidence in technology’s ability to offer remote services and an increased awareness of the limitations of traditional care models [9].

Recognising the potential of digital interventions in strengthening health systems, the World Health Organisation (WHO) emphasised their relevance in 2019 through its “Guidelines and Recommendations on Digital Interventions for Health System Strengthening” [10].

Innovative technologies in healthcare offer a practical solution to numerous systemic challenges, including rising costs, increased workloads for healthcare providers, and the shortage of healthcare professionals. They also facilitate the delivery of personalised services and enable the monitoring of patients beyond the conventional confines of healthcare settings, reducing the reliance on waiting medicine carried out in doctors’ offices or hospitals [11].

With these considerations in mind, healthcare professionals internationally have begun advocating for the virtual hospital model over the last decade. This model aims to provide specialised hospital-level care within communities, alleviating the strain on overwhelmed healthcare systems and achieving comparable, if not superior, clinical and health outcomes by integrating care models with an initiative medicine approach [12].

In the ever-evolving landscape of healthcare, the emergence of virtual hospitals will usher in a new era of patient care and accessibility [13]. The virtual hospital concept will prove to be a game-changer, providing remarkable benefits and advancements in delivering healthcare services [14, 15]. Long wait times, travel significant distances, or face geographical barriers to receive necessary medical care could be avoided with the development of virtual hospitals [16]. Virtual hospitals will be effectively transcending these limitations, bringing healthcare directly to patients’ fingertips [12]. Through the power of technology and telehealth capabilities, healthcare services could be available anytime, anywhere, offering unprecedented convenience and ease of access.

The concept of virtual wards, i.e., a part of a virtual hospital, has been around for quite some time [17], but during the pandemic, their utilisation was significantly enhanced, showing promising results in managing specific COVID-19 patients using pulse oximeters and secondary care monitoring. Consequently, substantial investments are now being channelled into expanding virtual ward capabilities, with a focus on accommodating frail patients. To assist integrated care systems and service providers in establishing and broadening virtual ward services, two key priority pathways have been introduced: acute respiratory tract infection virtual wards and hospital-at-home services for individuals with frailty [18, 19]. This represents a profound transformation in the delivery of healthcare for the elderly and is currently a top funding priority.

The impact of virtual hospitals extends beyond mere convenience. They could revolutionise the way healthcare is delivered, breaking down barriers and empowering patients with a personalized approach to their healthcare journey. From primary care to specialised consultations, mental health services to chronic disease management, virtual hospitals offer a comprehensive range of healthcare services that cater to the diverse needs of patients [20–22]. The objective of this narrative review is to explore the advantages and barriers of virtual hospitals in delivering healthcare, including access to specialized professionals, streamlined communication, efficient scheduling, integration of electronic health records, ongoing monitoring, and support, transcending geographical boundaries, and resource optimization.

## Methods

### Search Strategy

**FIGURE 1:**
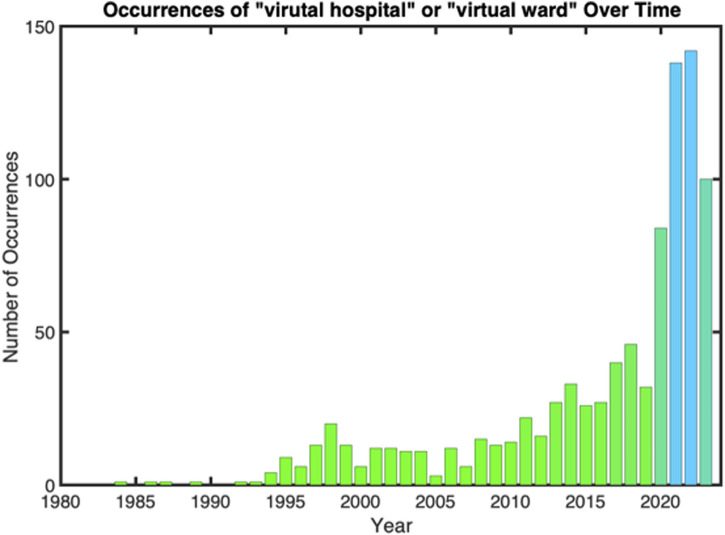
Occurrences of “virtual ward” and “virtual hospital” in the literature over time (Suresnes, France, 2024).

**FIGURE 2:**
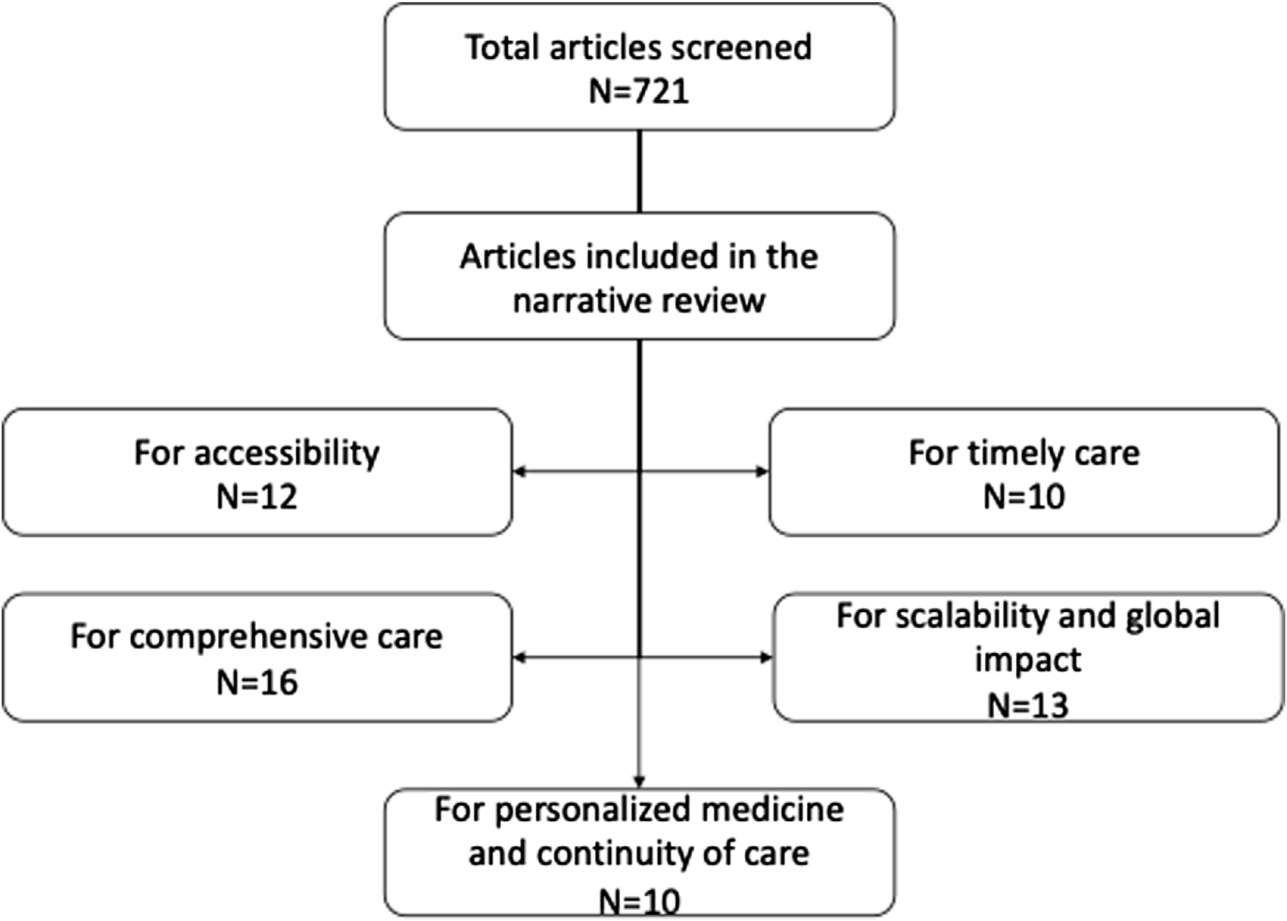
Flowchart (Suresnes, France, 2024).

PubMed Medline, Web of Science, Google Scholar, IEEE Xplore, and Scopus databases were used for the research, with only articles in English language, using the following terms: “virtual hospital” OR “virtual ward” (Figure 1). Articles included in this review were both, original research, reviews, systematic reviews and meta-analyses, viewpoints, and opinions articles. Literature was searched from inception to 2023 (Figure 2). The different outcomes for healthcare efficiency were defined based their interest in the literature for healthcare: accessibility [23, 24], comprehensive care [25], timely care [26], personalised medicine and continuity of care [27] and scalability and global impact [28, 29].

### Virtual Hospital

Fundamentally, a virtual hospital represents a healthcare institution that operates exclusively in the digital realm, devoid of a centralised physical presence. These virtual healthcare facilities are sometimes denoted as virtual care centres and are integral components of telehealth services. The Virtual Hospital model mirrors the conventional healthcare referral system. In this model, the patient’s medical information is gathered by e-clinics situated in remote third-world communities, utilising computers, or mobile phones. Subsequently, this data is transmitted to a general medical practitioner stationed at the virtual hospital, often referred to as the “Hub.” The general medical practitioner stationed at the Hub then proceeds to offer a diagnosis or directs the patient to the relevant virtual hospital department. These departments are interconnected through the Internet, enlisting specialist consultants from across the globe to collaborate on patient cases. The virtual hospital provides continuous remote assistance to patients, mirroring the care found in traditional hospitals. This centre doesn’t house any beds; instead, it exclusively accommodates healthcare professionals responsible for remote patient monitoring and virtual care delivery. To make this initiative successful, there’s a critical need for robust interoperability between various devices and information systems. Given that these virtual hospital care models aspire to deliver the same standard of care available in traditional hospitals, they function interconnectedly while maintaining a certain level of autonomy [30].

## Results

### Advantages

Virtual hospital can be characterised as “patient-centric” (Table 1). This designation stems from the continuous support provided by a collaborative healthcare team (team care) [31]. This approach emphasises care coordination and taps into the full spectrum of healthcare skills available within the healthcare system, ultimately enhancing patient-centred care. This, in turn, fosters greater patient autonomy and self-management [17]. Digital health monitoring plays a pivotal role in empowering patients and fostering co-production dynamics [32–34]. It enhances communication and relationships between patients and healthcare professionals, expedites diagnoses, enables more accurate interventions, and ultimately leads to an improved overall patient experience and outcomes. The virtual hospital model, thanks to its advanced digitalisation, allows for early disease identification and analysis, facilitating proactive interventions known as initiative medicine. This results in a deeper understanding of disease progression, leading to a significant reduction in mortality and a substantial enhancement in the quality of life [12]. The integration of virtual solutions and robotics can streamline administrative tasks, potentially reducing the risk of burnout of health professionals. This automation boosts staff efficiency and reliability while optimising operators’ time through more efficient workflow management for repetitive tasks and hospital support services [35]. Virtual hospitals contribute to cost reduction in healthcare by diminishing the need for hospital (re) admissions, conserving valuable resources, such as in-patient stays, and ensuring that patients receive the most appropriate care within the comfort of their homes [12].

**TABLE 1:** Advantages and Barriers for implementing virtual hospital (Suresnes, France, 2024).

Advantages	Barriers
Patients − Ongoing surveillance of chronic illnesses − Remote access to specialized services and expert guidance − Enhanced healthcare access and equity − Time and cost savings − Swift and precise diagnoses through tailored care plans and treatment profiles − Heightened predictability − Early detection and analysis of diseases − Fostered trust within the patient-health professional relationship − Elevated patient care experiences − Mitigation of aggravating factors and reduction in mortality − Diminished exposure to pathogens − Continuous care provided by a multidisciplinary team (team care) − Care delivery coordination with an Evidence-Based Medicine approach − Heightened autonomy and self-management verified by healthcare professionals, rather than self-referral − Improved patient-practitioner communication and relationships − Enhanced overall experiences and outcomes − Significant enhancement in quality of life Healthcare providers − Capacity to care for more patients − Reduction in waitlists − Resource conservation and sustainability improvement − Fewer emergency room admissions, hospital (re)admissions, and healthcare costs − Increased care efficiency and efficacy − Improved work-life balance for healthcare professionals, reducing burnout risks − Strengthened relationships and trust with patients, leading to fewer in-person consultations and hospital readmissions − Automation of repetitive tasks and hospital administrative and support services − Enhanced triage and organizational decision-making processes − Lowered error rates Health systems − Improved response to healthcare and social needs − Enhanced management of chronic conditions within communities − Superior health data sharing and information − More efficient allocation of resources and budgets − Enhanced quality and performance indicators for social and healthcare services	Infrastructure and Technology − Limited capacity for data collection and digitalization − Insufficient interoperability between devices and information systems − Absence of shared and interoperable standards for data digitalization − Lack of integrated data repositories (cloud-based and privacy considerations) − Uneven and fragile information technology infrastructure across the region − Limited adoption of Electronic Health Records Digital Competencies − Inadequate training in technology utilization − Scarce promotion of a digital culture − Shortage of experienced professionals in digital health, robotics, and automation Organization − Reluctance to embrace change and technological innovation − Complicated coordination among healthcare professionals with diverse skills − Complex regional organization and alignment with social needs − Necessity to revamp traditional care models with performance-driven logic and Diagnosis-Related Groups − Requirement to redefine career paths and roles of staff − Limited application to patient follow-up − Absence of common national objectives − Absence of a specific regulatory framework Culture − Low digital literacy − Restricted collaboration among stakeholders − Limited trust in digital health services from both clinicians and patients − Disruption of traditional value systems

### Barriers

To ensure the proper functioning of the system, cutting-edge technology and a robust, consistent information technology infrastructure must be universally accessible throughout the territory (Table 1). This system primarily relies on the collection, processing, and exchange of vast amounts of data. These data require meticulous collection and digitisation while adhering to precise, shared, and interoperable standards to guarantee their quality and interchangeability across various operators. Meeting this challenge necessitates collaborative efforts involving multiple stakeholders at both local and national levels. Conversely, the absence of shared standards poses a barrier to the uniform distribution of data across different regions. Consequently, this hinders the ability to compare medical, health, and epidemiological data, thereby impeding the seamless operation of artificial intelligence algorithms [36]. Currently, at the national level, there is a notable deficiency in the quantity and quality of collected and digitised data, along with a low level of interoperability among existing devices and operating systems. This deficiency is primarily attributed to the delayed digitisation of the sector, which, in part, results from the limited adoption of foundational tools like electronic medical records. The management and exchange of data may also raise concerns about privacy. Consequently, it is imperative to establish a specific regulatory framework. This framework would serve to clearly identify data ownership and define specific conditions that ensure the secure and ethical utilisation of data [37].

Efficient integration of healthcare data management offers the opportunity for caregivers to consolidate essential patient information within a unified database, enabling storage, assessment, and sharing with pertinent stakeholders [38]. Simultaneously, healthcare data management empowers medical experts to extract valuable insights for enhancing medical outcomes. Healthcare institutions are equally dedicated to upholding patient data privacy. Nevertheless, despite earnest endeavors, the healthcare sector has struggled to effectively maintain data security and protect privacy, resulting in the all-too-frequent occurrence of medical data breaches [39]. In addition, the healthcare sector bears the brunt of the financial burden, with data breaches incurring costs that are more than three times higher than those faced by other industries. It is imperative to recognize that data privacy and protection are fundamental rights of individuals [40]. Information amassed by healthcare organizations often includes patients’ personal information alongside medical data, and a data breach poses a significant threat to the confidentiality of this data, undermining the overarching purpose of digitalization in healthcare. Considering this, the imperative for robust data protection measures specific to the healthcare sector becomes apparent. Ransomware attacks in the healthcare sector predominantly exploit software vulnerabilities, employ phishing tactics, and target remote desktop protocol (RDP) as their primary modes of operation. Consequently, ensuring adequate data protection emerges as a paramount consideration prior to the integration of digital technology across all business sectors. The healthcare industry proves an ideal target for the theft of medical information, historically falling behind other leading sectors in safeguarding critical data [41]. Due to the relatively weaker security infrastructure within healthcare management, it stands as a vulnerable target on a global scale, presenting higher cybersecurity risks than other industries. This vulnerability is exemplified by the fact that, in the United States, approximately 81% of 223 surveyed organizations fell victim to cyberattacks, leading to the compromise of data belonging to over 110 million patients in 2015 alone [42]. Surprisingly, despite a 300% surge in cyberattacks over the past 3 years, only half of the healthcare providers in the United States believe they possess the capability to defend themselves effectively against these threats [42]. The primary factors that render the healthcare system an attractive target for cyberattacks are the wealth of information it holds and its susceptibility as a soft target.

## Discussion

### Current Examples of Virtual Hospitals

While this may appear idealistic today, it’s already a tangible reality in specific settings. For instance, Mercy Hospital in St. Louis, United States [43], it’s recognised as the world’s pioneering telemedicine centre and a “hospital without physical patients” [44]. At Mercy, in 2020, a team of 330 healthcare professionals, including doctors and specialized nurses, offers virtual healthcare services to a vast network of 600,000 patients spanning seven states, which include Arkansas, Kansas, Missouri, North Carolina, Oklahoma, Pennsylvania, and South Carolina. They operate from 160 workstations [45].

Australia, in 2020, has emerged as a trailblazer in the realm of virtual care. As a case in point, during the response to the COVID-19 crisis, the Sydney Local Health District effectively harnessed a virtual health service, known as Rpa-virtual. This service introduced an innovative care model characterised by its clinical rigor and safety, thereby enhancing the healthcare system’s resilience. The Rpa-virtual initiative paved the way for the establishment of a virtual-bed hospital, accommodating up to 2,000 Covid-infected patients. This virtual hospital was seamlessly integrated with the existing community nursing service, which cared over 1,000 patients [46].

In Brazil, strategies for remote healthcare were formulated and put into practice to ensure the continuity of HIV PrEP services for both adults and adolescents during the disruptions caused by the COVID-19 pandemic in 2020 [47]. At a major PrEP clinic located in Rio de Janeiro, telemedicine procedures encompassed the initial HIV rapid testing and telephone consultations, leading to the issuance of a digital prescription for a 120-day PrEP provision in addition to two HIV self-test kits. Subsequent teleconsultations were conducted from a distance through telephone calls, with the results of HIV self-tests transmitted via digital images. A cross-sectional, internet-based survey targeting PrEP users revealed a high level of satisfaction and acceptance with the teleconsultation and home delivery of PrEP services [48].

In Uganda, in 2021, the incorporation of telehealth services was a pivotal step in the HIV prevention and PrEP programs targeted at young women, ensuring uninterrupted service delivery amid the pandemic. These programs utilized “virtual safe spaces” for outreach and counseling, leveraging mediums like telephone, text messages, and WhatsApp. This approach was complemented by the home delivery of PrEP medications, HIV self-test kits, sanitary packs, and condoms, effectively ensuring the continuous provision of prevention services [49]. It is worth noting that PrEP home delivery was made available exclusively to individuals who did not require HIV testing (administered every 3 months), as HIV testing was still offered at healthcare facilities.

In Gambia, in 2020, patients with confirmed COVID-19 and who quality for virtual care were assigned a physician with a digital thermometer to monitor daily axillary temperature. A virtual plan monitor plan was performed by the physicians relied on mobile telephones and messaging applications such as WhatsApp [50].

In China, in 2020, the Zhongshan Ophthalmic Center (ZOC) of Sun Yat-sen University established a virtual clinical service using several digital technologies (5G telecommunication networks, big data analytics, artificial intelligence, and blockchain technology) to deliver online ophthalmic diagnosis and treatment services [51].

### Virtual Hospital for User Experience and Accessibility

Virtual hospitals prioritize user-friendly accessibility, offering straightforward navigation and secure telehealth technology for video consultations with healthcare professionals. They can ensure flexible appointment scheduling and compatibility with various devices, catering to a diverse population through multilingual support and accessibility features for individuals with disabilities. However, technical support teams are on hand for assistance, and educational resources empower patients to make informed healthcare decisions.

The virtual hospital experience is designed with a strong focus on user experience and accessibility, ensuring that patients can navigate the platform easily and access healthcare services seamlessly. Virtual hospitals prioritise user-friendly interfaces that are easy to navigate, even for individuals with limited technical skills [52]. The platform typically features clear and organised menus, intuitive icons, and straightforward instructions to guide patients through the various steps of their healthcare journey. Patients can easily streamline the appointment scheduling process through the virtual hospital platform, as it allows them to choose convenient time slots based on their availability. The platform features a user-friendly calendar interface that presents a clear display of available dates and times, minimising confusion and ensuring a seamless and hassle-free scheduling experience [53]. Robust and secure telehealth technology is employed by virtual hospitals to facilitate video consultations between patients and healthcare professionals [54]. The platform prioritises the privacy and confidentiality of medical information, implementing encryption and other security measures to ensure the secure transmission of data during video calls.

To enhance accessibility, virtual hospitals are designed to be compatible with a wide range of devices [55]. Whether patients prefer to use a computer, smartphone, or tablet, they can access the virtual hospital platform and engage in telehealth consultations from their device of choice, providing flexibility and convenience [56]. Recognising the diverse population it serves, virtual hospitals often offer multilingual support to cater to patients who are more comfortable communicating in their native language. This inclusivity allows individuals from different cultural backgrounds to access healthcare services and fully participate in their care [57]. Virtual hospitals aim to be accessible to individuals with disabilities by incorporating accessibility features [58]. This may include providing options for closed captions during video consultations, compatibility with screen readers for visually impaired patients, and ensuring the platform meets web accessibility standards to accommodate various disabilities [59]. In the event of any technical issues or challenges, virtual hospitals provide reliable technical support to assist patients [57]. Dedicated support teams are available to troubleshoot problems, guide patients through the platform, and address any concerns promptly, ensuring a smooth user experience [45]. To empower patients and enhance their understanding of their health conditions, virtual hospitals often offer educational resources within the platform. This may include access to articles, videos, or interactive tools that provide relevant information and support patients in making informed decisions about their healthcare [60–62].

### Virtual Hospital for Comprehensive Healthcare Services

Virtual hospitals may offer a wide range of healthcare services, including general check-ups, access to specialists, mental health support, diagnostic services, medication management, chronic disease care, remote monitoring, and wellness programs. Thus, patients can receive comprehensive care from the comfort of their own homes, making healthcare more accessible and convenient. Remote monitoring technologies could be utilized in some cases, and health promotion and wellness initiatives encourage patients to actively participate in their overall wellbeing.

Virtual hospitals serve as a primary point of contact for patients, providing services such as general check-ups, preventive care, and management of chronic conditions [63]. Patients can schedule appointments with primary care physicians who can address a broad range of healthcare needs, including routine examinations, minor illnesses, and health maintenance [64, 65]. Specialised medical expertise is easily accessible through virtual hospitals, as patients can connect with specialists from a wide range of fields, including cardiology, dermatology, neurology, orthopaedics, gastroenterology, and more [66]. This convenient access enables patients to receive expert opinions, second opinions, and personalised treatment plans without the need for physical referrals or extensive travel. The virtual platform brings the expertise directly to the patients’ screens, ensuring efficient and convenient healthcare delivery [67]. Recognising the importance of mental health, virtual hospitals often integrate mental health services into their offerings. Patients can access psychiatric consultations, counselling, and therapy sessions through video calls, ensuring that mental health support is readily available and accessible [68, 69]. Virtual hospitals facilitate diagnostic services, allowing patients to undergo necessary tests and receive accurate and timely results. This may include laboratory testing, radiology services, and even at-home diagnostic kits for certain conditions [12, 70]. The results can be securely accessed within the virtual hospital platform, enabling healthcare professionals to make informed diagnoses and treatment recommendations. In the realm of medication management, virtual hospitals offer a range of services that encompass prescription refills, adjustments, and medication reviews. Patients could consult with healthcare professionals to address their medications, potential interactions, and any concerns they may have [71]. This comprehensive approach guarantees that patients receive appropriate prescriptions and have uninterrupted access to the necessary medications for effectively managing their health conditions [71]. For individuals with chronic diseases, virtual hospitals offer comprehensive management programs. Patients can receive ongoing monitoring, personalised care plans, and education to effectively manage their conditions. Healthcare professionals collaborate with patients to optimise treatment, provide lifestyle recommendations, and offer support for self-management [72–74]. In certain cases, virtual hospitals utilise remote monitoring technologies to track patients’ health parameters from a distance. This allows healthcare professionals to remotely monitor vital signs, chronic disease indicators, and other relevant data. Patients may use wearable devices, mobile apps, or specialised equipment to transmit data securely to the virtual hospital platform, enabling healthcare professionals to monitor their progress and intervene as needed [75]. Virtual hospitals often emphasise health promotion and wellness initiatives. Patients can access resources, programs, and educational materials focused on lifestyle modifications, preventive care, nutrition, exercise, and stress management [76, 77]. This holistic approach encourages patients to take an active role in maintaining their overall health and wellbeing.

### Virtual Hospital for Efficient and Timely Care

Virtual hospitals can prioritize efficiency and timely care by leveraging user-friendly online platforms for appointment scheduling, reducing wait times by eliminating physical waiting rooms, and establishing efficient communication channels. They could ensure prompt delivery of test results and offer integrated prescription management systems for quick access to medications. Virtual hospitals facilitate referrals to specialists and further investigations directly within the platform, ensuring efficient coordination between healthcare providers. They also can focus on efficient follow-up care and monitoring, enabling timely adjustments to treatment plans and minimizing delays in ongoing care.

Virtual hospitals prioritise efficiency and timely care, leveraging technology to streamline the healthcare process and minimise waiting times. Virtual hospitals employ intuitive online platforms that allow patients to schedule appointments promptly [78]. Through user-friendly interfaces, patients can select available time slots that suit their schedule, eliminating the need for extensive phone calls or waiting for call-backs. This efficient appointment scheduling process ensures that patients can secure a consultation at their preferred time [22]. By eliminating the need for physical waiting rooms, virtual hospitals significantly reduce wait times for patients. Instead of spending hours in crowded waiting areas, patients can join their scheduled video consultations at the designated time, ensuring minimal delays. This improves overall patient satisfaction and allows healthcare professionals to adhere to their schedules more efficiently [79]. Efficient communication channels are established within virtual hospitals, allowing patients to easily connect with their healthcare providers through secure messaging systems. These streamlined channels enable quick and convenient exchanges of information, follow-up questions, and updates, eliminating the need for lengthy phone calls or email correspondences [80]. Virtual hospitals prioritise the seamless delivery of test results to patients. Diagnostic tests conducted remotely or in partner laboratories can be securely uploaded to the patient’s virtual health record. Patients can then access their results in real-time through the platform, eliminating the need for additional appointments or delays. This efficient delivery of test results enables healthcare professionals to promptly review and discuss the findings with patients [22, 81]. Integrated prescription management systems are often offered by virtual hospitals, allowing healthcare professionals to generate electronic prescriptions within the platform [71]. These prescriptions are immediately accessible to patients, who can conveniently retrieve them and obtain medications from local pharmacies without any unnecessary delays. This streamlined process ensures that patients receive timely access to the medications they need for their health conditions [82]. In cases where specialist consultations or additional investigations are required, virtual hospitals facilitate prompt referrals. Healthcare professionals can seamlessly refer patients to other specialists or order further tests directly within the virtual hospital platform [83, 84]. This enables efficient coordination between different healthcare providers, ensuring timely access to multidisciplinary care for patients with complex medical needs. Virtual hospitals prioritise efficient follow-up care and monitoring. Patients can easily schedule follow-up appointments, and healthcare professionals can track patients’ progress through regular virtual visits or remote monitoring technologies. This proactive approach enables healthcare professionals to address any concerns or changes in a timely manner, minimising delays in ongoing care [85].

### Virtual Hospital for Personalised Approach and Continuity of Care

Virtual hospitals can provide personalized healthcare with a focus on continuity. They maintain detailed patient profiles, fostering individualized care plans that consider unique needs, lifestyle factors, and preferences. These care plans encompass tailored treatment recommendations, medication adjustments, and preventive measures. Virtual hospitals ensure continuity of care by assigning primary healthcare providers and integrating electronic health records. They promote collaboration among healthcare providers, enabling a multidisciplinary approach to patient care. Patients benefit from continuous monitoring and support, including early detection of potential health issues.

Virtual hospitals are designed to provide a personalised approach to healthcare, ensuring that patients receive individualised care tailored to their unique needs. Additionally, virtual hospitals prioritise continuity of care to ensure a seamless healthcare journey for patients. Comprehensive patient profiles are established within the secure platforms of virtual hospitals. These profiles contain detailed information such as medical histories, allergies, medications, and previous treatments. With access to this information, healthcare professionals can gain a deeper understanding of each patient’s healthcare needs, empowering them to provide personalised care recommendations and develop tailored treatment plans. This ensures that patients receive individualised care that addresses their specific healthcare requirements [56]. Virtual hospitals foster strong patient-provider relationships by encouraging regular interactions between patients and healthcare professionals [86]. Through video consultations, patients can develop rapport, trust, and continuity with their healthcare providers. This personalised connection allows for open and honest communication, ensuring that patients feel heard and actively participate in their healthcare decisions [87]. Based on patients’ medical histories, symptoms, and goals, virtual hospitals create individualised care plans. These plans consider the specific needs and preferences of each patient, including lifestyle factors, cultural considerations, and treatment preferences. The care plans encompass personalised treatment recommendations, medication adjustments, lifestyle modifications, and preventive care measures, ensuring that the care provided aligns with the patient’s unique circumstances [88, 89]. Virtual hospitals prioritise continuity of care by ensuring that patients have consistent access to their designated healthcare teams. Rather than encountering different healthcare professionals at each visit, patients are often assigned a primary healthcare provider who oversees their care throughout their virtual hospital experience. This continuity ensures that patients receive consistent and coordinated care, enhancing the overall healthcare experience, and facilitating a deeper understanding of their medical conditions [90]. Electronic health records (EHRs) are seamlessly integrated into virtual hospitals, ensuring easy access to patient information. This integration provides healthcare professionals with a comprehensive view of each patient’s medical history, encompassing previous diagnoses, medications, test results, and treatments. By incorporating EHRs, virtual hospitals enable efficient and accurate decision-making, eliminate redundancies, and promote continuity of care across various healthcare settings [91]. This streamlined access to patient information enhances the overall quality and coordination of healthcare services. Virtual hospitals facilitate care coordination and collaboration among healthcare providers, ensuring a multidisciplinary approach to patient care. Healthcare professionals can easily communicate and share information within the virtual hospital platform, enabling a seamless exchange of knowledge and expertise. This collaboration allows for comprehensive treatment plans, shared decision-making, and a more holistic approach to patient care [92]. Patients receiving care through virtual hospitals benefit from continuous monitoring and support, extending beyond scheduled appointments [93]. This encompasses remote tracking of vital signs, utilisation of wearable devices to monitor specific health parameters, and the use of patient engagement tools that foster self-monitoring and self-care. Through proactive monitoring, virtual hospitals can detect potential health concerns at an early stage, enabling timely interventions or necessary support for patients. This approach ensures that patients receive ongoing attention and assistance, promoting their overall wellbeing and preventing potential complications [85].

### Virtual Hospital and the Potential for Scalability and Global Impact in Health

Virtual hospitals hold the promise of scalability and global impact in healthcare delivery. They transcend geographical boundaries, providing healthcare accessibility to underserved, remote, and resource-limited areas, reducing healthcare disparities, and enabling equitable access to services. By connecting patients with specialists worldwide, virtual hospitals offer a wider range of treatment options and improved health outcomes. In emergency response and disaster relief, they facilitate remote triage and initial medical guidance. Virtual hospitals can optimize resource utilization, efficiently allocate resources, and reduce healthcare costs while advancing telemedicine training and education, ensuring future healthcare professionals are well-equipped to utilize virtual healthcare platforms effectively.

One of the most promising aspects of virtual hospitals lies in their potential for scalability and global impact. Leveraging digital technologies and telehealth capabilities, virtual hospitals could revolutionise healthcare delivery on a global scale. By transcending geographical boundaries, virtual hospitals revolutionise healthcare accessibility, granting patients the ability to receive vital healthcare services irrespective of their physical location [94]. This is especially transformative for individuals living in underserved areas, remote regions, or areas with limited healthcare infrastructure. Virtual hospitals effectively bridge the gap between patients and healthcare providers, extending high-quality care to populations that previously encountered obstacles in accessing healthcare. As a result, individuals in remote or underserved areas can now benefit from the expertise and support of healthcare professionals, improving their overall health outcomes and wellbeing [12, 22, 94]. Virtual hospitals have the potential to reduce healthcare disparities by providing equitable access to healthcare services. By eliminating the need for physical travel, virtual hospitals empower individuals who may face challenges related to transportation, mobility, or financial constraints. This inclusivity ensures that individuals from all walks of life could receive timely and appropriate medical care [95]. By removing geographic barriers, virtual hospitals enable seamless access to specialised healthcare professionals regardless of location [66]. Patients can easily consult with specialists from around the world, eliminating the need for expensive and time-consuming long-distance travel. This not only reduces the financial burden but also opens up a wider range of treatment options. The ability to connect patients with experts in various fields holds great promise for improving health outcomes and expanding the scope of available treatments. Patients can now benefit from the knowledge and expertise of renowned specialists without the constraints imposed by distance, leading to enhanced healthcare experiences and better overall health outcomes [96]. Virtual hospitals play a vital role in emergency response and disaster relief efforts. During natural disasters or emergencies, traditional healthcare facilities may become overwhelmed or inaccessible [97]. Virtual hospitals can provide emergency telehealth services, allowing healthcare professionals to remotely triage, assess, and provide initial medical guidance to affected individuals. This capability can save lives, expedite care, and enhance disaster response efforts [97]. By maximising the utilisation of healthcare professionals and infrastructure, virtual hospitals offer scalability and resource optimisation [98]. Through efficient scheduling, virtual hospitals can effectively allocate resources, minimising idle time and optimising patient flow. This scalability enables virtual hospitals to handle increased demand and cater to a larger patient population without compromising on the quality of care. Additionally, by optimising resource utilisation, virtual hospitals have the potential to reduce overall healthcare costs, making healthcare more accessible and affordable for patients. The ability to scale efficiently and optimise resources is a key advantage of virtual hospitals, contributing to their effectiveness and cost-effectiveness in delivering healthcare services [99]. Moreover, virtual hospitals have the potential to advance telemedicine training and education [100, 101]. By incorporating telehealth technologies into medical education curricula, aspiring healthcare professionals can gain experience in remote consultations, telemonitoring, and tele-diagnosis [102]. This ensures that the next-generation of healthcare providers is equipped with the skills and knowledge necessary to leverage virtual healthcare platforms effectively.

In conclusion, virtual hospitals have emerged as a revolutionary approach to healthcare delivery, providing unparalleled convenience, accessibility, and personalised care. With their efficient and timely care, virtual hospitals could prioritise patient satisfaction and optimise health outcomes. Furthermore, their potential for scalability and global impact holds immense promise in addressing healthcare disparities and fostering international collaborations.
